# Compound 968 reverses adriamycin resistance in breast cancer MCF-7^ADR^ cells via inhibiting P-glycoprotein function independently of glutaminase

**DOI:** 10.1038/s41420-021-00590-1

**Published:** 2021-08-05

**Authors:** Ronghui Yang, Zihao Guo, Yiliang Zhao, Lingdi Ma, Binghui Li, Chuanzhen Yang

**Affiliations:** 1 Department of Biochemistry and Molecular Biology, School of Basic Medical Sciences, Capital Medical University, 100069 Beijing, China; 2 Advanced Innovation Center for Human Brain Protection, Capital Medical University, 100069 Beijing, China

**Keywords:** Breast cancer, Target identification

## Abstract

Adriamycin (ADR) is a chemotherapeutic drug widely utilized to treat multiple types of cancers; however, the clinical efficacy of ADR is compromised due to the development of drug resistance in patients. The combination of drugs with ADR may provide a better therapeutic regimen to overcome this obstacle. Glutaminase (GLS) has been explored as a therapeutic cancer target, and its inhibition also results in increased sensitivity of tumor cells to chemotherapeutic agents. This study aimed to investigate whether GLS inhibition could reverse ADR resistance. We treated the ADR-resistant MCF-7 (MCF-7^ADR^) cells with a GLS inhibitor, compound 968 or CB-839, in combination with ADR. We found that compound 968, rather than CB-839, together with ADR synergistically inhibited the cell viability. These results indicated that compound 968 reversed ADR resistance in MCF-7^ADR^ cells independently of GLS. Moreover, we modified the structure of compound 968 and finally obtained a compound 968 derivative, SY-1320, which was more potent than compound 968 in eliminating the drug resistance in MCF-7^ADR^ cells. Furthermore, using drug affinity responsive target stability and streptavidin–biotin immunoprecipitation assays, we demonstrated that SY-1320 could specifically target P-glycoprotein (P-gp) and increase ADR accumulation through inhibition of P-gp, thereby resulting in cell death in MCF-7^ADR^ cells. Together, our findings indicate that compound 968 or SY-1320 might be a promising drug for new combination chemotherapy in breast cancer to overcome the drug resistance.

## Introduction

Breast cancer, which has high mortality rates, is a frequently occurring cancer in women [1]. For early-stage breast cancer, surgical treatment has good therapeutic effects, while chemotherapy can significantly improve the 5-year survival rate of patients with middle- or advanced-stage breast cancer [2]. Among the chemotherapeutic agents, adriamycin (ADR) has been shown to have well-established benefits for these patients [3, 4]. It inhibits DNA topoisomerase II activity, forms adducts with DNA, and induces oxidative stress, thus mediating cell death [5, 6]. However, despite its success in improving cancer survival rates, long-term ADR treatment may lead to drug resistance and side effects [7, 8]. Increased expression of P-glycoprotein (P-gp), which is encoded by ATP-binding cassette subfamily B member 1 (*ABCB1*) gene, often contributes to ADR resistance in cancer [9, 10]. P-gp is a transport protein pump that has important roles in drug distribution and elimination. Many clinical trials using P-gp inhibitors are being evaluated, but the inhibitor toxicities or drug interactions drive researchers to develop more rational inhibitors [11–13].

A hallmark of cancer cell metabolism is the reliance on glutamine [14]. The glutamine metabolism depends on glutaminase (GLS), a rate-limiting enzyme that converts glutamine into glutamate. Thus, targeting glutamine dependence through GLS inhibition has emerged as an attractive strategy for cancer treatment [15]. A series of GLS inhibitors, such as compound 968, CB-839, and bis-2-(5-phe-nylacetamido-1,2,4-thiadiazol-2-yl)ethyl sulfide, have been studied [16]. Previous studies also found that GLS silencing led to the increased sensitivity of tumor cells to chemotherapy drugs. For instance, GLS inhibitor compound 968 could significantly sensitize ovarian cancer cells to paclitaxel [17]. The knockdown of GLS1 re-sensitized the taxol-resistant breast cancer cells to taxol [18]. However, whether GLS inhibition can reverse ADR resistance in ADR-resistant breast cancer cells remains unknown.

We found that a combination of ADR and compound 968, rather than CB-839, led to synergistic tumor cell death in MCF-7^ADR^ cells, suggesting that compound 968 might reverse ADR resistance independently of GLS. We also optimized the structure of compound 968 and obtained a derivative, SY-1320, which synergized with ADR to promote cell death more efficiently. Compound 968 or SY-1320 increased ADR accumulation and eliminated the ADR resistance in MCF-7^ADR^ cells through inhibition of P-gp. These results indicate that compound 968 or SY-1320 could function as a P-gp inhibitor to increase drug accumulation in MCF-7^ADR^ cells and represent a rational combination therapeutic strategy to overcome drug resistance.

## Results

### Compound 968 reverses the drug resistance in MCF-7^ADR^ cells

**Fig. 1:**
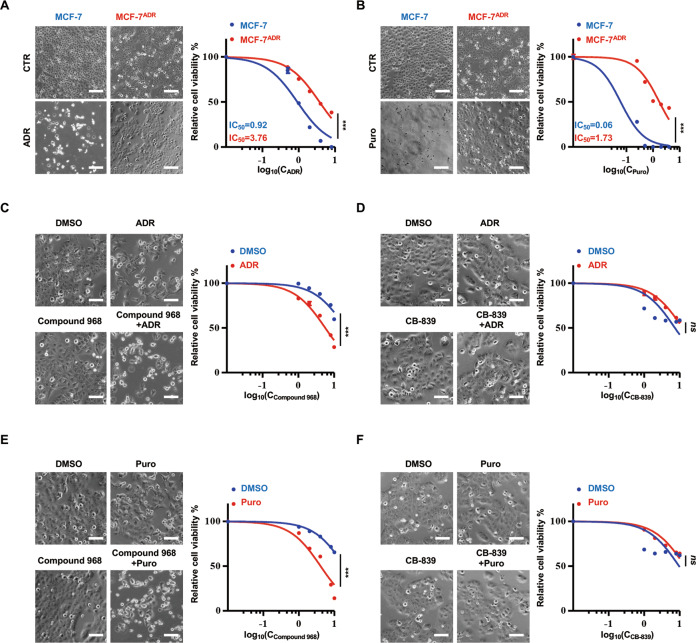
Combining compound 968 with adriamycin (ADR) or puromycin (Puro) results in decreased viability of MCF-7^ADR^ cells. **A**, **B** MCF-7 and MCF-7^ADR^ cells were treated with ADR or Puro as indicated for 48 h. Cell viability was assessed using the CCK8 assays, and the IC_50_ was calculated using GraphPad 7.0. Scale bars: 100 µm. **C–F** MCF-7^ADR^ cells were treated with compound 968 or CB-839 (0, 1, 2, 4, 8, and 16 µM) alone or in combination with 4 µM ADR (**C**, **D**)/2 µg/mL Puro (**E**, **F**) for 48 h. All groups were normalized to the non-inhibitor-treated group. Cell viability was assessed using the CCK8 assays. Scale bars: 50 µm. Data are presented as mean ± SD (three independent experiments). ns no significance; ***P < 0.001, one-way ANOVA.

The ADR-resistant cell line (MCF-7^ADR^ cells) was established by culturing MCF-7 cells with a low concentration and a continuous dose of ADR [19]. First, we verified the drug resistance of MCF-7^ADR^ cells to ADR. The sensitivity of MCF-7^ADR^ cells to ADR was greatly reduced compared to the cells without drug resistance induction (MCF-7; Fig. 1A). Long-term ADR induction often leads to multiple drug resistance. Indeed, MCF-7^ADR^ cells were also resistant to puromycin (Fig. 1B). Then we treated MCF-7^ADR^ cells with a GLS inhibitor (compound 968 or CB-839), together with ADR (or puromycin), to determine whether GLS inhibitors could reverse the drug resistance. Interestingly, compound 968, unlike CB-839, significantly promoted the sensitivity of MCF-7^ADR^ cells to ADR or puromycin. Moreover, compound 968, but not CB-839, when combined with ADR or puromycin induced apparent cell death (Fig. 1C–F). Taken together, these results suggest that compound 968 reverses ADR resistance in MCF-7^ADR^ cells independently of GLS.

### Optimizing the structure of compound 968 and screening more efficacious derivatives

**Fig. 2:**
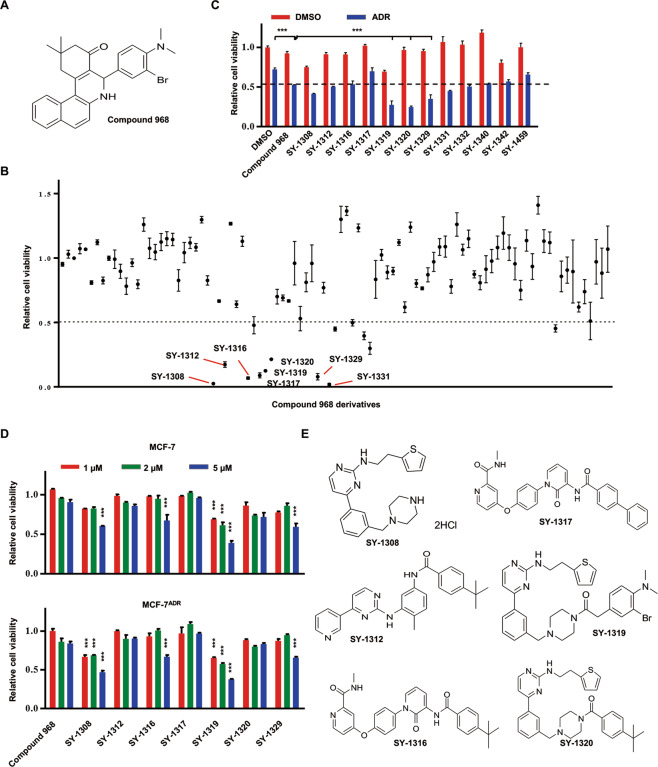
Compound 968 analogs exert synergistic inhibitory effects with ADR against MCF-7^ADR^ cell viability. **A** Chemical structure of compound 968. **B** Ninety-five analogs of compound 968 were synthesized and tested. MCF-7^ADR^ cells were treated with compound 968 analogs (10 µM) combined with ADR (2 µM) for 48 h. Cell viability was detected using the CCK8 assays. **C** MCF-7^ADR^ cells were treated with compound 968 or compound 968 analogs alone (2 µM), together with ADR (2 µM) for 48 h. Cell viability was detected using the CCK8 assays. **D** MCF-7 and MCF-7^ADR^ cells were treated with compound 968 or compound 968 analogs (0, 1, 2, and 5 µM) for 48 h. Cell viability was detected using the CCK8 assays. All groups were compared with the compound 968-treated group. **E** Chemical structures of compound 968 analogs. Data are presented as mean ± SD of three independent experiments. ***P < 0.001, two-tailed Student’s *t* test.

To optimize the combined effect of compound 968 and ADR, we modified the structure of compound 968 and obtained 95 derivatives (Fig. 2A, E). We found that 12 derivatives of compound 968 inhibited MCF-7^ADR^ cell proliferation by >50% at 10 µM when combined with 4 µM ADR (Fig. 2B). Next, we reduced the concentration of these derivatives to explore their synergistic effects with ADR. Among these compounds, several compounds were shown to be more potent than compound 968; SY-1320 exhibited stronger inhibition against MCF-7^ADR^ cell viability than compound 968 when combined with ADR (Fig. 2C). The cytotoxicity of SY-1320 alone to MCF-7 and MCF-7^ADR^ cells was moderate (Fig. 2D), indicating that SY-1320 reversed the ADR-insensitive phenotype of MCF-7^ADR^ cells.

### SY-1320, a compound 968 derivative, eliminates the drug resistance in MCF-7^ADR^ cells

**Fig. 3:**
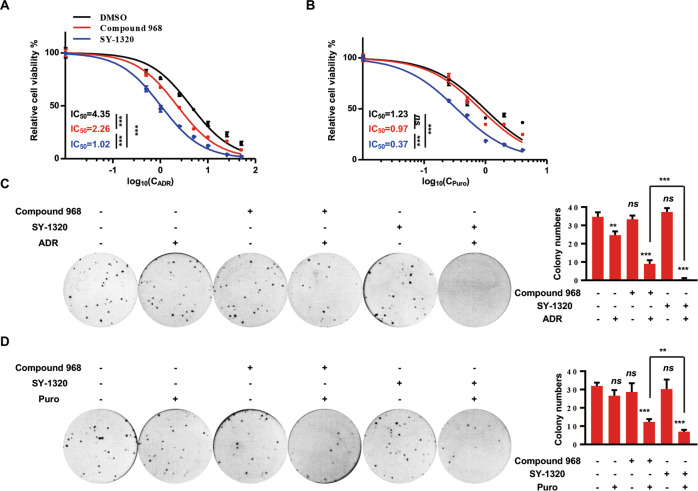
SY-1320 reverses multidrug resistance in MCF-7^ADR^ cells. **A**, **B** MCF-7^ADR^ cells were treated with a series of concentrations of ADR (**A**)/Puro (**B**) alone or combined with 2 µM compound 968 or SY-1320 for 48 h. Cell survival was measured using the CCK8 assays. **C**, **D** MCF-7^ADR^ cells were treated with the compounds as indicated for 2 weeks, and cell colonies were stained with crystal violet. In all, 1 µM compound 968, 1 µM SY-1320, 0.5 µM ADR, and 0.2 µg/mL Puro were used. Data are presented as mean ± SD of three independent experiments. ns no significance; **P < 0.01, ***P < 0.001, one-way ANOVA (**A**, **B**) and two-tailed Student’s *t* test (**C**, **D**).

To further confirm the synergistic inhibitory effects, MCF-7^ADR^ cells were treated with ADR in combination with compound 968 (2 µM) or SY-1320 (2 µM), as indicated for 48 h. The combination of ADR and compound 968 exhibited higher cytotoxicity (IC_50,_ 2.26 µM) than that of ADR (IC_50,_ 4.35 µM) alone in MCF-7^ADR^ cells (Fig. 3A). Moreover, SY-1320 significantly decreased the IC_50_ (1.02 µM) of MCF-7^ADR^ cells to ADR, which was similar to that of MCF-7 cells (IC_50_, 0.92 µM; Fig. 1A). The same results were obtained when the cells were treated with puromycin (Fig. 3B). Furthermore, the clonogenic survival assays showed that the combination of ADR and compound 968 (or SY-1320) significantly inhibited the colony formation, whereas ADR, compound 968 or SY-1320 alone had limited effects on the colony formation of MCF-7^ADR^ cells (Fig. 3C, D).

### Combination of SY-1320 and puromycin synergistically induces apoptosis in MCF-7^ADR^ cells

**Fig. 4:**
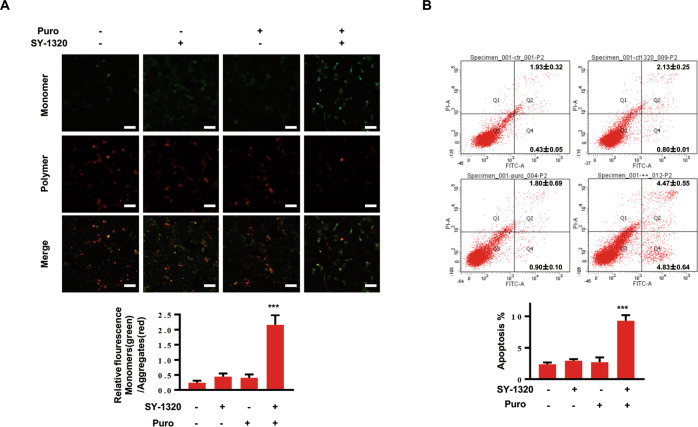
Combination of SY-1320 and puromycin synergistically induces apoptosis in MCF-7^ADR^ cells. **A** MCF-7^ADR^ cells were treated with Puro (1 µg/mL), SY-1320 (2 µM) alone, or both for 24 h, followed by JC-1 staining. Scale bars, 50 µm. **B** MCF-7^ADR^ cells were treated with Puro (1 µg/mL), SY-1320 (2 µM) alone, or both for 48 h. The cell apoptosis was detected by flow cytometry. Data are presented as mean ± SD of three independent experiments. ***P < 0.001, two-tailed Student’s *t* test.

ADR is a broad-spectrum anticancer drug that can induce apoptosis of a variety of tumor cells. However, the excitation spectrum of ADR overlaps significantly with that of propidium iodide. Thus, we used puromycin to determine whether the combination therapy could induce apoptosis. After treatment with puromycin (2 µg/mL), SY-1320 (2 µM), or both for 24 h, JC-1 staining was performed to detect the mitochondrial membrane potential. JC-1 is a monomer that cannot gather in the matrix of mitochondria and emits green fluorescence when the mitochondrial membrane potential is low. The results showed that the green fluorescence intensity in the combined group was significantly enhanced, while the red fluorescence intensity was weakened, suggesting that the membrane potential of mitochondria decreased after treatment with puromycin and SY-1320 (Fig. 4A). Decreased mitochondrial membrane potential is a crucial indicator of the early stage of apoptosis. We next detected the apoptosis of MCF-7^ADR^ cells after 48 h exposure to puromycin (1 µg/mL), SY-1320 (2 µM), or both by flow cytometry. The combination treatment resulted in apparent apoptosis in MCF-7^ADR^ cells, while the puromycin alone did not induce apoptosis (Fig. 4B). These results suggest that compound 968 could significantly eliminate the drug resistance in MCF-7^ADR^ cells, and SY-1320, a compound 968 derivative, has superior characteristics.

### P-gp is a target protein of compound 968 and SY-1320

**Fig. 5:**
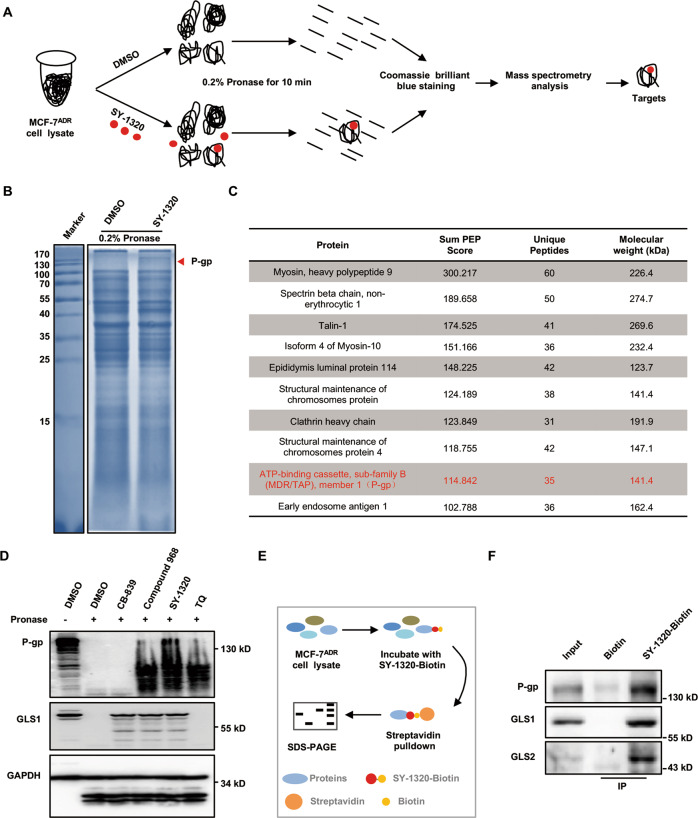
DARTS identifies that SY-1320 could target the multidrug resistance protein, P-gp. **A** Scheme of DARTS. **B** MCF-7^ADR^ cell lysates were incubated with DMSO or SY-1320 (1 mM) for 2 h at 4 °C and then subjected to pronase digestion for 10 min, after which Coomassie blue staining was performed. **C** Mass spectrometric results showed that P-gp might be a potential target for SY-1320. **D** Direct binding between P-gp and compound 968 or SY-1320 was identified by DARTS. Tariquidar (TQ, a P-gp-specific inhibitor) served as a positive control. **E** Scheme of Streptavidin–biotin immunoprecipitation. **F** Streptavidin–biotin immunoprecipitation of the interactions between SY-1320 and its targets.

We used a previously reported method, drug affinity responsive target stability (DARTS) [20, 21], to further elucidate how compound 968 and SY-1320 eliminated the drug resistance in MCF-7^ADR^ cells, to identify the target protein of SY-1320 (Fig. 5A). The cells were lysed, and then the lysates were divided into two equivalent volumes. The samples were incubated with dimethyl sulfoxide (DMSO) or SY-1320 (1 mM) and then proteolysed with 0.2% pronase for 10 min, followed by Coomassie blue staining. We observed a strong protective band at 120–300 kDa in the SY-1320-treated group, whereas no detectable band was noted in the DMSO-treated group (Fig. 5B). We next identified the proteins by mass spectrometry and ranked the results using the Sum PEP Score (Fig. 5C). Among the top 10 candidates, P-gp (or MDR1, encoded by multi-drug resistant gene *ABCB1*) alone was shown to be related to the drug resistance in tumors. Indeed, we found the proteolytic protection effects of P-gp in the compound 968- or SY-1320-treated groups, whereas no such effects were observed in the DMSO- or CB-839-treated groups (Fig. 5D). Furthermore, we verified the results of mass spectrometry using the streptavidin–biotin immunoprecipitation assays (Fig. 5E). GLS1 and GLS2 are targets for compound 968, thus served as the positive control. SY-1320 bound to P-gp, as well as GLS1 and GLS2 (Fig. 5F). Together, these results indicate that P-gp is a target protein of compound 968 and SY-1320.

### Compound 968 and SY-1320 enhances ADR accumulation in MCF-7^ADR^ cells by inhibiting P-gp

**Fig. 6:**
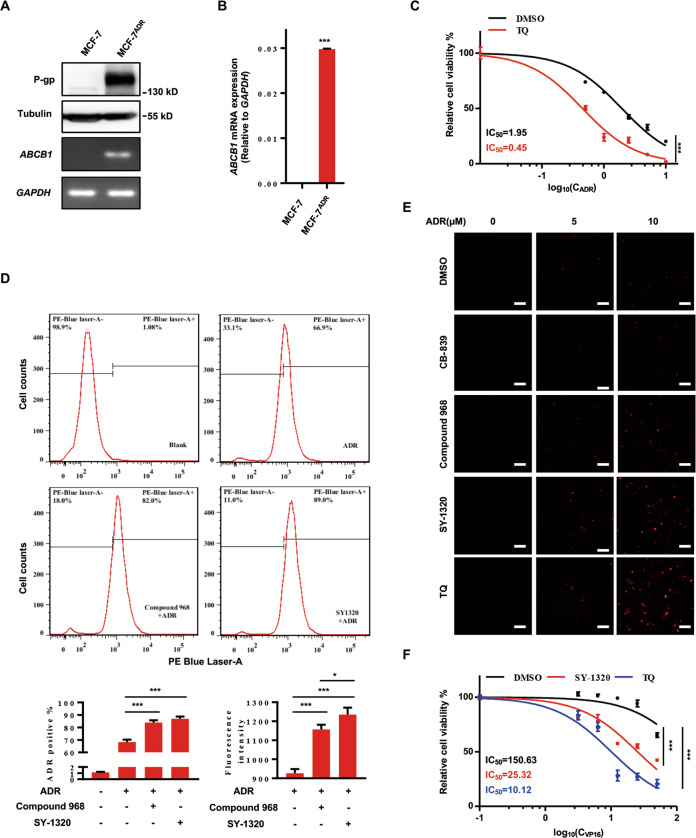
SY-1320 promotes ADR accumulation in MCF-7^ADR^ cells by targeting P-gp. **A**, **B** Levels of P-gp protein and mRNA in MCF-7 or MCF-7^ADR^ cells were detected by western blot and RT-qPCR, respectively. **C** MCF-7^ADR^ cells were treated with ADR (2 μM) alone or together with TQ (0.5 μM) for 48 h. Cell viability was detected using the CCK8 assays. **D**, **E** Intracellular ADR accumulation was detected by flow cytometry (**D**) and fluorescence (**E**). Scale bars, 50 µm. **F** Both SY-1320 and TQ increased the sensitivity of MCF-7^ADR^ cells to etoposide (VP16). Data are presented as mean ± SD of three independent experiments. *P < 0.05, ***P < 0.001, two-tailed Student’s *t* test.

As P-gp is the target protein for SY-1320, we speculated that SY-1320 might eliminate the drug resistance in MCF-7^ADR^ cells by inhibiting P-gp. We first measured the expression of P-gp protein and mRNA in MCF-7 and MCF-7^ADR^ cells and showed that the expression of P-gp protein and mRNA levels were higher in MCF-7^ADR^ cells than that in MCF-7 cells (Fig. 6A, B). Then we treated MCF-7^ADR^ cells with tariquidar (TQ), a reported P-gp-specific inhibitor, and also observed significant combined inhibitory effects with ADR, which were similar to that of SY-1320 or compound 968 (Fig. 6C). P-gp can actively pump hydrophobic antitumor drugs out of the cell, thus reducing drug accumulation and increasing drug efflux. We then determined whether ADR accumulated in MCF-7^ADR^ cells. The percentage of ADR-positive cells significantly increased in the combination treatment groups compared to the ADR treatment group (Fig. 6D). Notably, the fluorescence intensity of ADR in cells was also enhanced when treated with the combination of compound 968 or SY-1320 (Fig. 6E, TQ as a positive control and CB-839 as a negative control). Taken together, compound 968 and SY-1320 promoted the accumulation of ADR in MCF-7^ADR^ cells by inhibiting P-gp. Moreover, we also used another antitumor drug, etoposide (VP16), to determine whether MCF-7^ADR^ cells developed multidrug resistance (Fig. 6F). MCF-7^ADR^ cells were also insensitive to VP16, and SY-1320 reversed the resistance of MCF-7^ADR^ cells to VP16. Together, these results indicate that compound 968 (or SY-1320) could serve as a P-gp inhibitor to increase drug accumulation in multidrug-resistant cells.

## Discussion

The overexpression of P-gp or other ATP-binding cassette (ABC) transporter family members usually causes multidrug resistance [22]. Therefore, the inhibition of P-gp might be a feasible treatment strategy to overcome multidrug resistance. In the past decades, many inhibitors targeting P-gp have been found and tested in clinical trials; however, the P-gp inhibitors have not achieved widespread acceptance due to severe side effects or a lack of efficacy [23–28]. Despite unexpected clinical trials outcomes, P-gp remains to be a crucial target for overcoming the obstacles of multidrug resistance. This study showed, for the first time, that compound 968, a GLS1 inhibitor, could function as a P-gp inhibitor to increase drug accumulation in multidrug-resistant cells.

Wang et al. first identified compound 968 as a GLS inhibitor [29]. They screened the small-molecule inhibitors and found that compound 968 could significantly inhibit the activity of Rho GTPases, which was indirectly caused by the inhibition of GLS activity. In addition, they showed that treatment with 10 µM compound 968 had no effects on normal NIH3T3 cells, suggesting that compound 968 had low toxicity to normal cells. We demonstrated that 4 µM compound 968 was sufficient to suppress the activity of P-gp and reversed multidrug resistance. Our observations indicate that compound 968 has a strong inhibitory effect on the activity of P-gp at a safe dose, and compound 968 might be a promising P-gp inhibitor.

Among the 95 derivatives of compound 968, SY-1320 was the most potent compound that suppressed the survival of MCF-7^ADR^ cells when used in combination with ADR treatment. SY-1320 was also more potent than compound 968. Further results demonstrated that the inhibition efficiency of SY-1320 on P-gp was high, but the inhibitory effect on GLS still needs to be verified. Most of the effective derivatives had partially similar structures, thus providing a reference for the optimization and development of new P-gp inhibitors (Fig. 2).

Cancer cells exhibit fundamentally altered cellular metabolism to support cell growth. The high demand for glutamine is one of the characteristics of many tumor cells [30, 31]. The glutamine metabolism is initially catalyzed by GLS, which converts glutamine into glutamate. GLS activity has a correlation with the proliferation of cancer cells, implying that GLS has the potential as a druggable target [32]. Several GLS inhibitors have been developed based on the crucial functions of GLS in various cancer types that are driven by the dysregulated glutamine metabolism [33]. CB-839 is the best-studied GLS inhibitor and is currently being evaluated in clinical trials [34–36], whereas studies about compound 968 are limited. We showed that compound 968, unlike CB-839, acts as a P-gp inhibitor, thus suggesting that compound 968 might be used in combination with anticancer drugs to overcome the drug resistance. The inhibitory effect of compound 968 on GLS and P-gp also makes it superior to other P-gp inhibitors in killing cancer cells dependent on glutamine metabolism.

Overall, our study suggested that compound 968 or its derivative SY-1320 significantly eliminated multidrug resistance in MCF-7^ADR^ cells. Compound 968 or SY-1320 could increase the drug accumulation in MCF-7^ADR^ cells by suppressing P-gp. Thus, this combination strategy might be potentially used for treating drug-resistant tumors, especially those dependent on glutamine metabolism.

## Materials and Methods

### Cell culture

The human MCF-7 cell line was purchased from ATCC and was used to develop the ADR-resistant MCF-7 (MCF-7^ADR^) cell line via exposure to ADR (2 µM). All the cells were cultured in Dulbecco’s modified Eagle’s medium (DMEM; Gibco, Rockville, MD, USA) with 10% fetal bovine serum (Gibco, Rockville, MD, USA) and 1% streptomycin and penicillin (Gibco, Rockville, MD, USA) at 37 °C in an atmosphere of 5% CO_2_. The MCF-7 was recently authenticated by short tandem repeat analysis and tested negative for mycoplasma. ADR, compound 968, CB-839, and TQ were purchased from MedChemExpress (Monmouth Junction, NJ, USA) and dissolved in DMSO.

### Compound library and screening

A total of 95 compound 968 analogs were synthesized by Centaurus BioPharma Co., Ltd (Beijing, China). Stock solutions of all of the compound 968 analogs were prepared in DMSO (10 mM) and stored at −20 °C. MCF-7^ADR^ cells were plated on 96-well plates (2000 cells per well) in triplicate. The cells were incubated with ADR (2 µM) combined with DMSO or the library compounds (10 µM) in the library after 24 h. The number of cells was determined using the Cell Counting Kit-8 (MedChemExpress, Monmouth Junction, NJ, USA) assays 48 h later. The compounds that inhibited the cell viability of MCF-7^ADR^ by >50% were selected as the candidates.

### Western blotting

The protein was extracted by RIPA buffer and the concentration was measured by bicinchoninic acid (BCA) assay (Beyotime, Shanghai, China). Sodium dodecyl sulfate–polyacrylamide gel electrophoresis (SDS-PAGE) was performed with the same amounts of proteins. After blocking, the polyvinylidene difluoride membrane was incubated with the primary antibodies as follows: P-gp (22336-1-AP, Proteintech, USA), GLS1 (12855-1-AP, Proteintech), glyceraldehyde 3-phosphate dehydrogenase (GAPDH; 10494-1-AP, Proteintech), and GLS2 (ab113509, Abcam, UK).

### Cell apoptosis assay

The MCF-7^ADR^ cells were plated on six-well plates with DMEM medium. One day later, the cells were treated with puromycin (1 µg/mL) alone or in combination with SY-1320 (2 µM). Cell apoptosis was measured using JC-1 staining (MedChemExpress, Monmouth Junction, NJ, USA) or an apoptosis detection kit (Beyotime, Shanghai, China).

### Clonogenic survival assay

The drug cytotoxicity of the indicated compounds was evaluated by the clonogenic assays. Briefly, MCF-7^ADR^ cells (300 cells per well) were plated on 6-well plates and treated with ADR (2 µM) or puromycin (1 µg/mL) alone or in combination with SY-1320 (2 µM). After 2 weeks, crystal violet staining was performed, and the clones were counted.

### DARTS and mass spectrometry

MCF-7^ADR^ cells were lysed with lysis buffer containing the phosphatase and protease inhibitors. After centrifugation (4 °C, 14,000 r.p.m.,15 min), the concentration of protein was detected. Then 120 µg of protein was added into the reaction buffer (50 mM Tris-HCl [pH 8.0], 10 mM CaCl_2_, and 50 mM NaCl) to make a final volume of 100 µL. Then DMSO or SY-1320 (1 mM) were incubated with the mixture on ice for 30 min. All steps were performed on ice to ensure the stability of protein. After quickly warming to room temperature, the samples were proteolysed with 0.2 µg of pronase (Sigma-Aldrich, St. Louis, MO, USA) for every 100 µg of protein for 10 min. Then 20 µL 6× protein loading buffer was added to the samples to stop the reaction. The proteins were separated using SDS-PAGE gel, after which the gels were stained with SimplyBlue Coomassie (Invitrogen, Carlsbad, CA, USA), and specific bands were cut out and subjected to mass spectrometric analysis.

### Streptavidin–biotin immunoprecipitation

MCF-7^ADR^ cells were harvested and lysed using RIPA buffer and EDTA-free Protease Inhibitor Cocktail (Roche, Germany). After pre-clearing, the concentration of protein was detected. The cell lysate with 500 µg of proteins was incubated with biotin or SY-1320-biotin at 4 °C for 2 h. After adding the streptomycin-coated beads, the samples were rotated at 4 °C for 2 h. The beads were washed with RIPA buffer three times, after which the biotinylated drug–protein complex was separated from the beads with 20 μL of 10 mM biotin for 30 min. Finally, equal volumes of 2× protein loading buffer were added to the samples, and proteins were separated using SDS-PAGE gel.

### Reverse transcription (RT) and real-time PCR

RNA was extracted from the cells using TRIzol Reagent (Sigma-Aldrich, St. Louis, MO, USA). Then 1 µg of total RNA was reverse-transcribed to cDNA using a FastQuant RT Kit (Tiangen, Beijing, China). The primers for ABCB1 and GAPDH were as follows: ABCB1 forward, TGACAGCTACAGCACGGAAG and reverse, GCCATCAAGCAGCACTTTCC; and GAPDH forward, GTCAGCCGCATCTTCTTT and reverse, CGCCCAATACGACCAAAT. Fast Start Essential DNA Green Master (Roche, Germany) was used to perform quantitative real-time PCR.

### Intracellular Adriamycin accumulation

MCF-7^ADR^ cells were plated on six-well plates with DMEM. The cells were treated with DMSO, ADR (2 µM), and ADR (2 µM) combined with SY-1320 (2 µM) or compound 968 (2 µM) 24 h later. The intracellular ADR accumulation was detected by BD LSRFortessa flow cytometry.

### Statistical analysis

The experiments were repeated at least three times. The statistical analysis was conducted using Student’s *t* test (two-tailed) and one-way analysis of variance. All results are shown as mean and standard deviation, and *P* values < 0.05 indicated a significant difference. Analysis and graphical presentation were performed using the SPSS 19.0 software (Chicago, IL, USA) and GraphPad 5.0 software (San Diego, CA, USA).

## Data Availability

All data generated or analyzed during this study are included in this published article.
